# A Case of Atrial Fibrillation and Rapid Ventricular Response in a 22-Year-Old Athlete With Sickle Cell Trait

**DOI:** 10.7759/cureus.95437

**Published:** 2025-10-26

**Authors:** Tristan S Moseley, Steven M Sasser, Ryan T Jordan, Matthew D Overturf

**Affiliations:** 1 Medicine, Edward Via College of Osteopathic Medicine, Monroe, USA; 2 Anatomical Sciences, Edward Via College of Osteopathic Medicine, Monroe, USA

**Keywords:** atrial fibrillation (af), sickle cell disease (scd), sickle cell trait, supraventricular tachycardia (svt), synchronized cardioversion

## Abstract

Sickle cell trait (SCT) is generally regarded as a benign carrier state of sickle cell disease (SCD); however, emerging evidence indicates that it may be associated with adverse cardiovascular outcomes, including atrial fibrillation (AF). AF is uncommon among young, otherwise healthy athletes, and its occurrence in individuals with SCT is not extensively documented. We present a case involving a 22-year-old male collegiate football player with a history of SCT who presented with palpitations initiated during practice. Upon arrival, he was hemodynamically stable; nonetheless, his initial electrocardiogram (ECG) revealed atrial fibrillation with rapid ventricular response (AF RVR). Laboratory investigations were unremarkable, including a normal troponin level. The patient was administered intravenous (IV) diltiazem for rate control, and synchronized cardioversion was successfully performed to restore sinus rhythm. Short-term anticoagulation therapy with apixaban was prescribed, along with flecainide on an as-needed basis for rhythm management. The patient was subsequently referred for outpatient catheter ablation and placed on a three-month restriction from contact sports. This case underscores a rare presentation of AF RVR in a young athlete with SCT. Exertional stress may contribute to arrhythmogenesis through mechanisms such as vaso-occlusion, hypoxia, acidosis, and dehydration. Current guidelines recommend rate control, cardioversion when clinically indicated, and short-term anticoagulation in cases of new-onset AF. Catheter ablation presents a potential definitive therapeutic option for young, symptomatic individuals. Preventive measures for athletes with SCT should include adequate hydration, heat acclimatization, and education of players and coaching staff. This case highlights the importance of heightened awareness, adherence to guideline-based management, and the necessity for further research into the association between SCT and AF in young athletes.

## Introduction

Sickle cell trait (SCT) constitutes a heterozygous, often asymptomatic carrier state of sickle cell disease (SCD). Individuals with SCT inherit a single defective hemoglobin S (HbS) gene alongside one normal copy of hemoglobin A (HbA). Typically, these individuals do not exhibit signs of sickling and generally have a life expectancy comparable to that of the general population [1]. Conversely, SCD remains the most common inherited blood disorder worldwide, affecting approximately 100,000 individuals in the United States (US) [2]. Although clinical presentations can differ, more severe manifestations include acute pain crises, acute chest syndrome, and significant organ damage. Cardiopulmonary complications are the primary cause of mortality among patients with SCD and are associated with an increased risk of atrial fibrillation (AF). AF is the most prevalent arrhythmia observed in athletes across all age groups, with a prevalence ranging from 0.3% to 12.3% [3]. Established risk factors for AF within this demographic include chronic kidney disease (CKD), left atrial dilation, left ventricular hypertrophy, and hypertension [4]. Recent research has identified an association between SCT and adverse cardiovascular and renal outcomes. Large cohort studies have demonstrated a correlation between SCT and an increased risk of AF, although causality has not yet been confirmed [4]. This risk may be amplified in athletes with SCT who undergo excessive heat exposure and dehydration, leading to a reduction in blood pH levels [5].

SCT can also impact young, otherwise healthy individuals. Between 1974 and 2010, there were 18 documented fatalities attributable to sickling among college football players. In each instance, the athletes were engaged in strenuous activity at the time of their death. Subsequent investigations revealed that athletes with SCT faced a 37-fold increased risk of exertional death compared to those without SCT. Patients experiencing exertional sickling report mild pain and progressive muscular weakness until their legs are unable to support their own body weight [6].

The relationship between SCT and AF has not been comprehensively clarified in existing scholarly literature. A proposed hypothesis suggests that SCT may predispose individuals to mild renal dysfunction, thereby promoting inflammation and activation of the renin-angiotensin-aldosterone system (RAAS) [7]. The resulting renal insufficiency could lead to cardiac remodeling and arrhythmias such as AF [6,8]. However, this association remains correlative, and direct causality within SCT has not been definitively established.

## Case presentation

We report a 22-year-old male patient with a past medical history significant for sickle cell trait. The patient presented to the emergency department with reports of palpitations that commenced during football practice. He observed that his heart rate increased significantly while running. He denied experiencing dizziness, lightheadedness, shortness of breath, or chest pain. The patient noted a mild symptomatic improvement since arriving at the emergency department. Vital signs (Table 1) were taken and blood drawn for complete blood count (CBC) and comprehensive metabolic panel (CMP) analysis (Table 2).

**Table 1:** Observed vital signs

Vital Sign	Value
Blood Pressure	117/87 mmHg
Pulse	172 bpm
Respiration Rate	15 breaths/min
O_2_ Saturation	97%

**Table 2:** Complete blood count and comprehensive metabolic panel results MCV: mean corpuscular volume; MCHC: mean corpuscular haemoglobin concentration; RDW: red cell distribution width; BUN: blood urea nitrogen; BNP: brain natriuretic peptide; TSH: thyroid-stimulating hormone

Complete Blood Count (CBC)		
Parameter	Measured Value	Reference Value
WBC	6000 cells/mcL	4,000-10,000 cells/mcL
RBC	6.01 million cells/mcL	4.5-6.1 million cells/mcL
Hemoglobin	15.9g/dL	13-17 g/dL
Hematocrit	48.90%	40-55%
MCV	81 fL	80-100 fL
MCHC	32.5 g/dL	27-31 g/dL
RDW	12.70%	12-15%
Platelets	272,000 cells/mcL	150,000-400,000 cells/mcL
Comprehensive Metabolic Panel (CMP)		
Sodium	142 mEq/L	135-145 mEq/L
Potassium	4.0 mEq/L	3.7-5.2 mEq/L
Calcium	9.8 mg/dL	8.5-10.2 mg/dL
Glucose	77 mg/dL	60-100 mg/dL
Chloride	110 mEq/L	96-106 mEq/L
Creatinine	1.24 mg/dL	0.6-1.3 mg/dL
BUN	19 mg/dL	6-20 mg/dL
Protein Total	8.1 g/dL	6.0-8.3 g/dL
Albumin	4.7 g/dL	3.4-5.4 g/dL
CO_2_	24 mmol/L	23-29 mmol/L
Other Parameters Measured		
High Sensitivity Troponin	<3 ng/L	<14 ng/L
BNP	13 pg/mL	<100 pg/mL
Magnesium	1.9 mg/dL	1.7-2.2 mg/dL
TSH	2.915 uIU/mL	0.4-4.5 uIU/mL
Free T4	1.09 ng/dL	0.8-1.9 ng/dL

**Figure 1:**
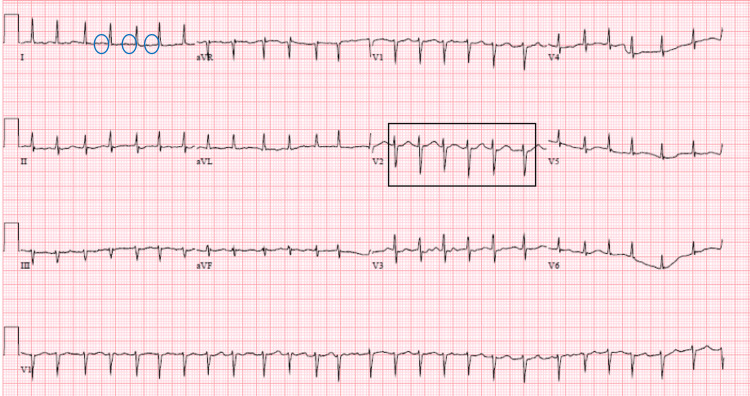
A 12-lead ECG depicting atrial fibrillation with rapid ventricular response Atrial fibrillation is characterized by the absence of P waves (indicated by blue circles). Additionally, the presence of rapid ventricular contractions is observed, as denoted by the segment within the black box.

A 12-lead electrocardiogram (ECG) demonstrated atrial fibrillation with rapid ventricular response (AF RVR), with rates fluctuating between 130 and 155 bpm (Figure 1).

At this juncture, cardiology was consulted, and the patient was administered 20 milligrams of intravenous diltiazem, subsequently initiating a continuous infusion of 10 mg per hour. Shortly thereafter, AF RVR was converted to supraventricular tachycardia (SVT), and a bedside transthoracic echocardiogram (TTE) was performed to evaluate structural and functional integrity. The TTE findings were not available for review. Synchronized electrical cardioversion was subsequently conducted, and a subsequent 12-lead ECG confirmed the re-establishment of normal sinus rhythm. Cardiology advised that the patient be prescribed apixaban for a duration of one month, along with as-needed flecainide at discharge to manage any breakthrough recurrences. It was also recommended that the patient avoid contact sports during this period, pending outpatient atrial fibrillation ablation with pulmonary vein isolation via radiofrequency energy or pulsed field ablation (PFA). The patient has remained symptom-free since this episode and is currently awaiting ablation as a definitive treatment.

## Discussion

To the best of our knowledge, this is a rare presentation of a young football athlete with SCT experiencing new-onset AF RVR during physical exertion. The patient received diltiazem, which facilitated the conversion to SVT, subsequently reverting to normal sinus rhythm following electrocardioversion. It should be noted that, due to the absence of an ECG demonstrating SVT, the rhythm must be considered speculative. As a definitive, long-term intervention, the patient will undergo catheter ablation, a procedure that involves delivering thermal or electrical energy to generate small lesions in targeted cardiac regions, predominantly around the pulmonary veins. This scar tissue effectively disrupts abnormal electrical pathways, thereby preventing recurrence of atrial fibrillation [9].

This case should raise suspicion that extreme exertion in patients with SCT can provoke similar complications observed in SCD. Repetitive exertion and subsequent exhaustion may result in vaso-occlusion, severe metabolic acidosis, hypoxia, and dehydration. A prior study on exertional sickling demonstrated that acidosis increases the propensity of red blood cells to undergo irreversible sickling at a partial pressure of oxygen (PO_2_) of 25 mmHg in vitro [10]. A lack of consensus remains regarding the management of exertional collapse associated with sickle trait (ECAST). The National Athletic Trainers’ Association (NATA) has issued a position statement proposing a framework to differentiate between sickling-related collapses and other diagnoses, enabling appropriate response by athletes and staff (https://www.nata.org/). Various organizations, including the National Collegiate Athletic Association (NCAA) and the American Orthopedic Society of Sports Medicine (AOSSM), have referenced NATA’s recommendations. To mitigate risk, it is advised that all athletes undergo SCT testing, as recommended by the NCAA, if they have not been previously tested. Emphasis should be placed on exertional acclimatization, avoiding excessive heat exposure, and ensuring adequate hydration for athletes with SCT. Furthermore, all coaches, players, and trainers should be acquainted with established prevention and treatment protocols. It should be noted that the patient was slightly hyperchloremic (110 mEq/L). This was the only electrolyte abnormality detected, likely attributable to dehydration resulting from football practice [11].

The current guidelines issued by the American Heart Association (AHA) recommend the use of either a beta-blocker or a non-dihydropyridine calcium channel blocker, such as intravenous diltiazem, administered to patients with both acute and chronic AF RVR, provided the ejection fraction (EF) exceeds 40%. It is additionally advised to administer an initial bolus of diltiazem, followed by a continuous infusion [12]. There are no differences in this treatment approach for young, otherwise healthy patients with SCT. Diltiazem acts by slowing conduction through the atrioventricular (AV) node, thereby extending the refractory period of the AV node. Evidence suggests that it achieves ventricular rate control more effectively than metoprolol and digoxin when administered as a bolus [13]. Following the administration of intravenous diltiazem, the patient’s rhythm transitioned to SVT, necessitating synchronized cardioversion. The 2015 AHA Guideline for the Management of Adult Patients with Supraventricular Tachycardia recommends that synchronized cardioversion be performed in patients with SVT who do not respond to intravenous beta-blockers, diltiazem, or verapamil, or when these treatments are not feasible [14,15]. Prior to cardioversion, clinicians should consider performing an echocardiogram to evaluate cardiac structure and function and to assess for thromboembolism. Both the AHA and the American College of Cardiology advocate for TTE in all patients with AF.

For patients presenting with a shorter duration of AF (<48 hours), the risk of thromboembolism is low, and anticoagulation prior to cardioversion may not be necessary. In this case, the patient had a CHA_2_DS_2_-VASc score of 0 and subsequently underwent synchronized cardioversion in the emergency department [16]. Anticoagulation may still be initiated with a CHA_2_DS_2_-VASc score of 0 in patients with SCT or SCD due to their risk of transient ischemic attacks (TIAs). CHA_2_DS_2_-VASc factors in patient age, sex, history of congestive heart failure, hypertension, diabetes, stroke, thromboembolic events, and vascular diseases [15]. Since SCT and SCD are hematologic genetic disorders, they are generally not included in the calculation of CHA_2_DS_2_-VASc scores [17]. This suggests a more precautionary approach to the administration of oral anticoagulation within this patient population.

Following synchronized electrical cardioversion, the patient achieved resolution of AF and returned to normal sinus rhythm. He was provided with a one-month supply of oral apixaban, a reversible factor Xa inhibitor, as atrial fibrillation significantly elevates the risk of stroke by nearly fivefold, and most patients require prophylactic anticoagulation [16]. The patient was also prescribed as-needed flecainide, a class IC anti-arrhythmic agent utilized for AF and paroxysmal SVTs. Flecainide functions by blocking fast-inward cardiac sodium (Na^+^) channels, thereby prolonging depolarization and increasing refractory periods [18]. Patients with structurally normal hearts on this medication should also be on an atrioventricular (AV) nodal blocker to prevent 1:1 conduction if atrial flutter occurs [19].

Patients undergoing cardiac ablation typically experience a three-month recovery period, commonly referred to as the “blanking period.” During this phase, inflammation and scar tissue formation occur as part of the healing process. Since healing is not complete during this period, recurrent arrhythmias are not considered indicative of procedural failure [18]. Consequently, although the duration of recovery remains a subject of debate, athletes with atrial fibrillation (AF) should refrain from contact sports for at least three months post-ablation. Return to sport is deemed appropriate by a cardiologist when there are no symptomatic episodes following successful ablation, a normal heart rhythm is restored, the risk of recurrence is low, and anticoagulation therapy has been discontinued [20,21].

As previously noted, it is exceedingly uncommon for an individual with SCT to exhibit symptoms. This case illustrates that the rare occurrence of AF RVR may manifest in otherwise healthy young adults, a demographic typically not regarded as high risk for arrhythmias. The diagnostic and therapeutic decisions involved in managing these patients highlight significant clinical considerations, particularly in situations where guidelines offer limited direction for this age group. Future research should explore the under-researched association between SCT and atrial arrhythmias, as well as develop more definitive protocols for prevention and education targeting athletes, coaches, trainers, and healthcare providers. Furthermore, research should be undertaken to investigate the conversion to SVT following the administration of diltiazem, as this represents a rare and inadequately documented phenomenon.

## Conclusions

AF, although exceedingly rare in young adults, may manifest in athletes with SCT, and exertion should be recognized as a potential precipitant for arrhythmia within this demographic. While catheter ablation may provide a definitive solution in selected cases, management should adhere to established guidelines, including rate control, synchronized electrical cardioversion, and short-term anticoagulation depending on stroke risk or uncertainty of AF onset, as the preferred approach for patients experiencing new-onset AF RVR. Further research is essential to elucidate the association between SCT and AF among young athletes engaged in contact sports, thereby supporting the development of more targeted management strategies for high-risk patients.
